# Multi-ethnic computational thinking and cultural respect in unmanned aerial vehicle-assisted culturally responsive teaching

**DOI:** 10.3389/fpsyg.2023.1098812

**Published:** 2023-03-10

**Authors:** Ying-Hsun Lai

**Affiliations:** Department of Computer Science and Information Engineering, National Taitung University, Taitung, Taiwan

**Keywords:** unmanned aerial vehicle, culturally responsive teaching, computational thinking, cultural respect, multi-ethnic learning effectiveness

## Abstract

**Introduction:**

As education systems worldwide begin to accept and implement computational thinking, the educators of both elementary and higher education are considering the cultivation of students’ computational thinking abilities. It is hoped that students effectively analyze and deconstruct all kinds of complex issues with computational thinking, and seek computer-executable ways to solve real-world problems. Through the integration of program education, students can learn and develop the abilities to practically apply their theoretical learning in information technology education. With the promotion of the concept of multicultural education, an increasing number of educational arenas are gradually introducing the concept of multicultural education to inculcate in students respect for different ethnic cultures via multicultural integration.

**Methods:**

In this study, unmanned aerial vehicle (UAV) technology was used to introduce culturally responsive teaching. The objective was to build a UAV-assisted culturally responsive teaching environment for multi-ethnic students that is based on their different thinking mechanisms formed by their respective cultures and living environments. Multi-ethnic students can attempt to solve problems employing computational thinking that is implemented when programing to control UAV. With the influence of culturally responsive teaching, the UAV-assisted learning strategies helped students and teachers of multi-ethnic groups understand different cultures and learn through mutual aid and cooperation.

**Results and Discussion:**

This study discussed the computational thinking abilities via different dimensions: logical thinking, programming ability, and cultural respect. The results showed that the introduction of UAV-assisted culturally responsive teaching method benefits not only indigenous students. For Han Chinese students as well, owing to the influence of cultural understanding, their overall learning effectiveness and cultural respect will be strengthened. Thus, this method improves the learning effectiveness in programming of multi-ethnic students, as well as of students with weaker prior programming ability. The method can also enhance the cognition and comprehension of different cultures in multicultural education.

## 1. Introduction

Computational thinking effectively analyzes and deconstructs complex issues before converting them into computer programming languages, so that people can understand human problems through computer programming and use computers to implement solutions (Wing, 2006, 2011; Cansu and Cansu, 2019). Application of computational thinking does not imply that humans are expected to think like computers; it involves the solving of complex human problems using a gamut of requisite thinking tools (Lavigne et al., 2020; Lai et al., 2021). The computational thinking process comprises four steps—problem decomposition, model identification, abstraction, and algorithm design—which attempt to transform various problems into a form that can be addressed using computers or machines. Adapting to the development needs of students in the information age, the International Society for Technology in Education (ISTE) specially provided a definition of computational thinking competency standard, which includes algorithmic thinking, creativity, logical thinking, and problem-solving skills. This demonstrates that, in addition to imparting basic programming skills to students, computational thinking education emphasizes cultivating their problem comprehension skills, system design skills, and ability to solve real-world problems. Therefore, computational thinking is suitable for applying the basic literacy and thinking methods of various stages and fields to integrate cross-field applications and is suitable for course teaching in different fields (Qualls and Sherrell, 2010; Del Olmo-Muñoz et al., 2020; Relkin et al., 2021). Currently, computational thinking has been introduced in other courses. Subjects such as mathematics, science, social studies, language, and arts have their respective learning behavior objectives when integrating the concept of computational thinking ability. Several studies focus on the application of such concepts by holding different types of activities based on programs, to strengthen learners’ reading ability and enable them to write using programming languages and perform computational thinking, and introducing training on programming so that students can learn through osmosis and improve their ability to apply what they have learned in information technology education. In practice, UAV can be controlled by programs, which facilitate students’ acquisition of relevant technical knowledge and training in the basics of programming; accordingly, the tools have become a common teaching and learning aid in technology-assisted education (Molina et al., 2014; Baca et al., 2021; Bolick et al., 2022; Shadiev and Yi, 2022).

As Taiwan is located between the Eurasian continent and the Pacific Ocean, throughout its 400-year history, it has been ruled consecutively by different countries and ethics. Thus, Taiwan has developed into a country with multi-racial integration. In addition to the various cultures brought to Taiwan by people from different countries, the Taiwan indigenous people also form a unique, diverse, and colorful part of Taiwan’s cultural palette. These people comprise 16 ethnic groups, including Amis, Atayal, Paiwan, and Bunun, each with its unique cultural characteristics because of different histories and geographical environments. The number of tribes in Taiwan is even larger—above 700. Indigenous people from different ethnic groups living in different geographical locations and having different degrees of Sinicization display different customs and cultures, which results in diverse cultural characteristics. Each ethnic group has its cultural argument and perception. Considering the trend of the expansion of globalization and Taiwan’s history and geography, it has become inevitable for researchers to consider multicultural integration, and there is a need to implement multicultural teaching as the concept of multiculturalism has been extended to the field of education (Cobern and Loving, 2001; Menkel-Meadow, 2018). Multicultural education is an essential part of the puzzle of Taiwan’s education. According to Taiwan’s Curriculum Guidelines, a basic principle of classroom teaching activities is that most students should be able to participate and be integrated; this allows them to experience the nation’s cultural diversity and gain the intercultural competence of respecting differences while pursuing substantial equality in the education process, so that multicultural values can be protected (Nesterova and Jackson, 2018; Hasanah et al., 2021). A key factor in multicultural education is respect and acceptance of diverse cultures (Yılmaz and Boylan, 2016). Banks (2021) presented a more detailed explanation of multicultural education, indicating that it is an act of educational reform and the implementation process of changing the educational structure. Instead of affecting only students, the courses, teachers, schools, or even educational institutions have to agree on and fully implement the objectives of multicultural education. The study also proposed five dimensions of multicultural education, which include the integration of learning content, the process of knowledge construction, prejudice reduction, an equity pedagogy, and empowering school culture. Through the five dimensions, a positive and diverse school culture can be created, so that different ethnic groups can develop well (Banks and Banks, 2001).

In addition to identifying and respecting cultural values, multicultural education also focuses on alleviating the negative influence on the learning effectiveness of ethnic minorities by ensuring that they receive the same education as the majority in schools and are not affected by the fact that the teaching materials are designed for the majority. In many countries worldwide, different approaches for improving teaching according to the characteristics of cultures and ethnic groups have been established (Liang and McQueen, 1999). However, in Taiwan, indigenous people have long been bound by geographical and economic constraints. They usually live in remote areas, and it is difficult for them to travel to other areas, which results in the scarcity of economic resources and insufficiency of cultural influence. Indigenous peoples can also be further subdivided into mountain indigenous peoples and plains indigenous peoples—the former face a greater problem of information gap. Additionally, owing to the given environmental factors, the intention and effectiveness of indigenous students in acquiring information and programming skills are often weaker (Liu and Lin, 2010). Qualitative interviews have revealed that this is because of students’ insufficient understanding of the effects of learning programming on their future, as well as a low tribal self-awareness. Respondents usually see no direct benefit and influence of programming education on their future career or tribal development. Additionally, as indigenous students usually have weaker foundations in digital skills compared with ordinary Han Chinese students, they are unwilling to learn programming, and therefore generally perform poorer in the subject. Many indigenous students even consider suspending their education as they feel anxious about the fact that passing the information and programming courses is necessary for graduation, and they are apprehensive that they may have to delay graduation to complete these courses (Tsai et al., 2022). However, as indigenous students in Taiwan possess fewer living resources and are disadvantaged in terms of cultural education, they have a more urgent need to improve their science and technology literacy. In addition to acquiring the abilities to practice, master, and control the development of science and technology, surmounting their constraints in employment, and enhancing employability with relevant technological capabilities, what is crucial is alleviating the difficulties faced by each tribe and fostering the development of the whole tribe by considering their unique problems based on their thinking ability with respect to technology and the cultivation of self-creative characteristics.

The main purpose of this research is to establish a multicultural teaching design and enhance the self-identity of indigenous students in science and technology. This study proposes the use of unmanned vehicles to introduce culturally responsive teaching in the exploration of computational thinking research. Culturally responsive teaching is supported by the aid of unmanned aerial vehicle technology, and students can consider the application of the Han Chinese culture and indigenous cultures to unmanned vehicles during the design process. Multi-ethnic students can understand the establishment of a multicultural educational environment through culturally responsive teaching activities and solve problems through computational thinking. It can promote indigenous students’ intentions to learn and understand programming, as well as improve their execution effectiveness, while displaying their advantages in affection and craftsmanship through the implementation of teaching activities so that indigenous students’ confidence in the field of information can be enhanced; simultaneously, students from both the Han Chinese and indigenous cultures can develop mutual cultural respect.

## 2. Literature review

### 2.1. Computational thinking education

The concept related to computational thinking first appeared in 1980 when Seymour Papert proposed that computers can promote thinking and explained how children can use computers to change their approach to acquiring knowledge and expressing their own ideas (Papert, 1980). This concept was widely noticed in 2006 when Wing (2006) extended its application to problem-solving. Subsequent studies recommended the introduction of computational thinking to various fields of educational research as a core skill to learn in K-12 education, and develop teaching methods for it (Wing, 2006, 2008, 2011). With the recent popularity of artificial intelligence (AI) technology in programming education, the focus on scientific and technological literacy has gradually transformed into technological programming literacy, which stimulates students’ creativity, enhances their self-learning and logical thinking abilities, and cultivates their computational thinking and problem-solving abilities (Voskoglou and Buckley, 2012; Yadav et al., 2016). Given the increasingly complex problems of daily life, computational thinking is used to effectively analyze complex issues, solve problems with the process of computational concepts, as well as analyze and understand the problem-solving behaviors of humans (Wing, 2006; Román-González et al., 2017). Computational thinking ability can be subdivided into the following four dimensions:

- Decomposition: Clearly define the problem and decompose it into smaller, individual problems.
- Pattern Recognition: Identify specific patterns or observe similarities in the data.
- Abstraction: Convert data or actions into abstract data or display them in codes.
- Algorithm design: Design a corresponding algorithm or use program codes to describe and implement actions.

By successively nurturing the abilities related to these four dimensions, computational thinking can be effectively cultivated in a phased manner (Wing, 2008). Computational thinking, which is effective in analyzing and solving problems, is increasingly explored by educators and researchers as it is considered a key competency that enables students to master the fundamental skills of problem-solving (Weintrop et al., 2014). In K-12 education, students’ computational thinking literacy is mainly developed by the program training method (Wei et al., 2021). The use of computational thinking can enable students to master higher-order thinking processes, such as deconstructing problems and thinking creatively (Shute et al., 2017). The ability of computational thinking is not only applicable to the research on K12 education, but it can also be used in the field of higher professional education (Tang et al., 2020). In professional fields, computational thinking can be used to help analyze and solve problems, so as to improve the motivation for program learning, while students who are not majoring in information can easily understand how programs operate and become more interested in acquiring programming skills (Aoki et al., 2013). Different subjects, including mathematics, robotics, and music, or the integration of the current Internet of Things (IoT) and AI technology learning, can be used in computational thinking education to foster students’ ability (Bell and Bell, 2018). Many studies have also developed related technological tools, such as augmented reality (AR), virtual reality (VR), and even robotic aids to assist computational thinking education (Chen et al., 2017; Lin et al., 2021).

Barr and Stephenson (2011) subdivided computational thinking ability into various dimensions; they introduced the concept to other subjects such as mathematics, science, social science, languages, and arts to discuss the corresponding learning behavior objectives when integrating computational thinking ability. A few studies focus on the application of computational thinking by holding several types of activities based on programming, to strengthen learners’ reading and reading abilities, and enable them to write using programming languages and practice computational thinking (Román-González et al., 2017). Additionally, several studies have combined ubiquitous learning with the thinking ability of computational thinking, which extends the application to improving students’ ability to use computers in their work, instead of solely training them in coding (Tsai et al., 2017). The cultivation of computational thinking skills reveals that students can use such scientific methods to locate the key parts of problems and seek solutions using programs, and this process can effectively increase their interest in practicing programming (Hambrusch et al., 2009). Additionally, when students subdivide a problem into smaller, easier-to-solve problems, their interest in learning the research topic—and even their learning effectiveness—can be maintained, as they can gain a sense of accomplishment by locating the key issues in the process of solving each small and detailed problem (Qualls and Sherrell, 2010). Barack Obama had proposed a plan to cultivate the basic programming skills of everyone. A study has revealed that the introduction of the concept of computational thinking facilitates the easy comprehension of students who are not majoring in information regarding how programs operate, and thereby gain interest in programming (Aoki et al., 2013). The further development of technical expertise and career interests through the combination of different fields has also led to a higher diversification of computers and even the combination of current IoT applications and AI technology learning.

### 2.2. Culturally responsive teaching

According to the definition of anthropology, culture is a combined consensus of a specific group of people on values, cognition, code of conduct, beliefs, and customs, and material lifestyles, whereas multiculturalism is a criticism of the situation that liberalism emphasizes equality between ethnic groups but neglects cultural differences, so that the mainstream culture swallows or rejects weak cultures, resulting in their disadvantaged status (Young, 2005). What multiculturalism advocates is more than universal equality and anti-assimilation. A crucial point to be emphasized is the recognition of each other’s cultural differences, so that different ethnic groups can have the right to retain their exclusive cultural characteristics in the spirit of recognition (Wilkesmann et al., 2009). As an island country with a territory that is narrow and long, people from various countries have migrated to Taiwan; however, its indigenous people display the most abundant and diverse cultures (Chang and Ding, 1995; Fujita et al., 2013). Indigenous people from different ethnic groups living in different geographical locations and having different degrees of Sinicization display different customs and cultural practices, which results in diverse cultural characteristics. Taiwan’s indigenous people usually live in remote locations and are economically disadvantaged; the unique features of their cultures create a distance from the Han Chinese culture. In many studies related to the learning characteristics of indigenous people, their unsatisfactory academic performance has been attributed and categorized into “cultural factors,” “socioeconomic statuses of parents,” “racial discrimination issues,” “education funding issues,” “teacher qualification issues,” and “the issues of students’ ability,” which are regarded as the most significant factors affecting their performance (Maseleno et al., 2016). Among them, cultural issues are due to the fact that educational courses and teaching materials are still tailored to mainstream culture, mostly ignoring the needs of indigenous peoples. When participating in such courses, indigenous students have a sense of estrangement and uncertainty about the future, because they cannot understand how learning the courses would lead to the realization of their self-worth and self-definition; this usually results in negative emotions toward learning and poor academic performance. The establishment of multicultural education is a key factor in solving this problem, and the only way for indigenous students to identify with the courses is to establish a diverse education design that integrates tribal experience and values into the whole culture of indigenous peoples and includes these features into the design of education (Sylva et al., 2010).

Culturally responsive teaching is a method of teaching practice developed for multicultural education; its overall concept relates to the use of the background, cultural characteristics, prior knowledge, and even life experience of multi-ethnic students as teaching materials or approaches for learning. Ethnic characteristics can serve as a reference, whereas the diverse cultures of students can serve as a bridge in teaching. Culturally responsive teaching can help improve the motivation and performance of minority students or of students with unsatisfactory performance. Its connotation includes four aspects: building tolerance, developing attitudes, enhancing meaning, and cultivating ability. However, in the multicultural education of Taiwan, the courses that consider indigenous students’ ethnical backgrounds mostly belong to the fields of social studies and languages; science subjects have not been practicing the same level of culturally responsive teaching. Studies that promote the integration of diverse cultures into science education and the 12-year compulsory education have been emerging and encourage different ethnic groups and communities to design multicultural courses according to their cultural tendencies (Feng et al., 2013; Liu, 2016). It is necessary for multicultural teaching to use culture, language, and multicultural courses in parallel so that different ethnic groups and communities can have equal opportunities to participate in various activities, such as those related to culture, which enable them to retain their own cultural characteristics while cultivating respect for others. Relevant studies have built mathematical models of ingenious peoples and discussed their applicability; the processes were guided by five stages: “preparation and planning,” “exploration and understanding,” “reading and design,” “review and revision,” and “practice and reflection.” Additionally, investigation work including observation, interviews, questionnaire surveys, and collection and analysis of relevant documents was conducted (Brown, 2017). Teachers are the main entities for implementing educational plans in culturally responsive teaching. Influenced by their own cultures or education, teachers usually design teaching materials based on personal cognition or teach according to the customary cognition of the majority and neglect the educational cognition of the minority. Regarding the construction of teaching materials, Gay and Howard (2000) advocated that students’ own culture should be used for thinking and bridging ideas, so that students can understand the teaching content based on their self-awareness, previous experience, or knowledge systems, which can improve their overall motivation and learning effectiveness. The study proposed four major concepts of culturally responsive teaching: the power of caring, culture, and communication in the classroom, ethnic and cultural diversity in curriculum content, and cultural congruity in teaching and learning. Additionally, the content of teaching is not the only focus of culturally responsive teaching; it also emphasizes mutual respect among students despite their differences, so that cross-cultural teaching can be conducted in a safe learning space where teachers and students accept each other. The ultimate goal is to enhance students’ motivation to learn, as well as establish multicultural equality and just responses (Howard, 2003; Roll et al., 2019). Regarding culturally responsive teaching, the overall literature mainly suggests that the backgrounds and experiences of multicultural students should be considered in the teaching process so that they can build a bridge of knowledge. A teaching environment that allows diverse cultures to blend should also be created so that knowledge can be disseminated by designing multicultural teaching materials and teaching activities that are centered around students, to enhance their overall learning motivation and effectiveness.

## 3. Research methods

### 3.1. Research model

**Figure 1:**
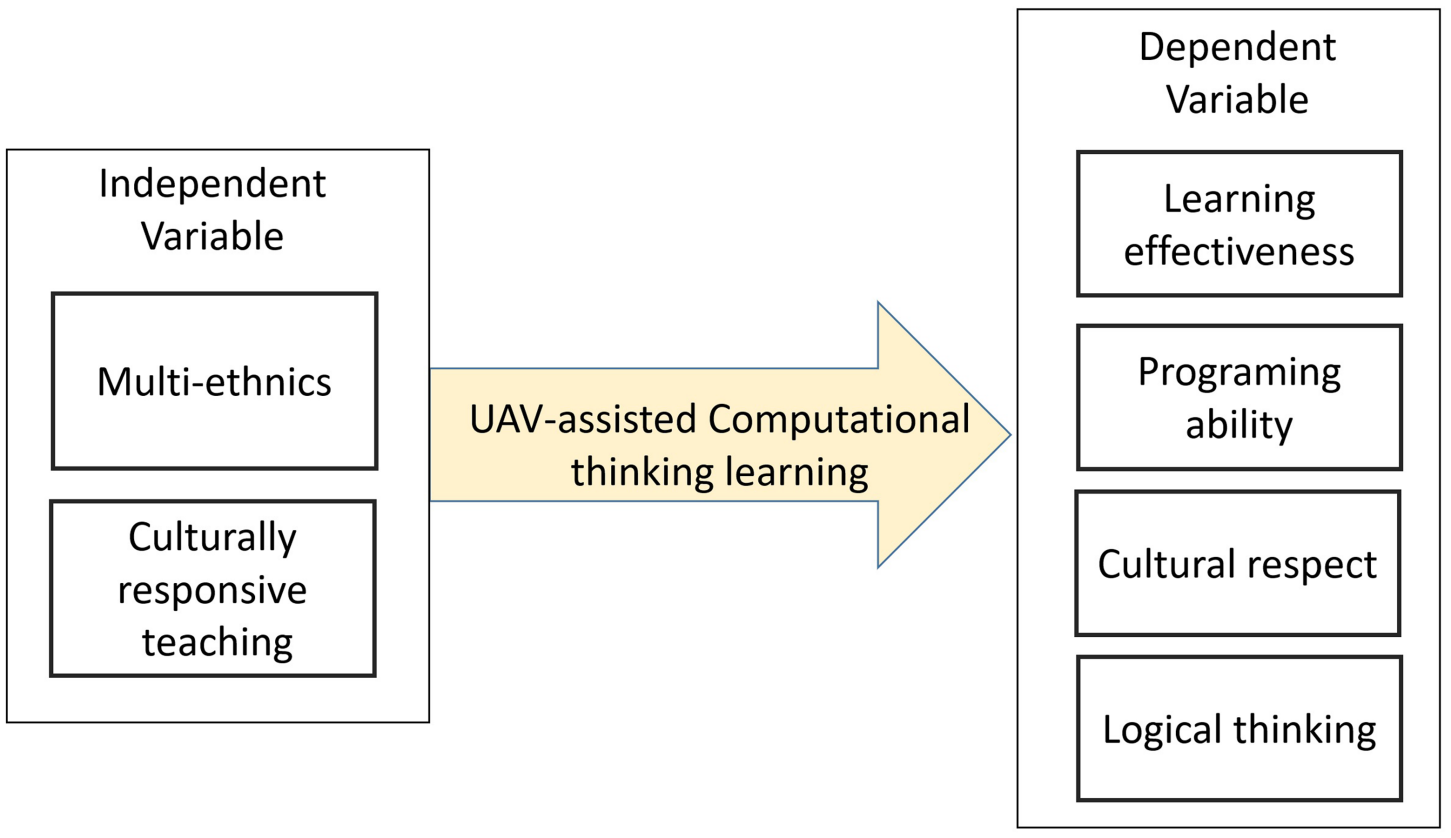
Research Model.

This study explores how unmanned vehicles can be integrated into culturally responsive teaching and reflects on the computational thinking performance of Han Chinese students and indigenous students. It aims to construct a teaching method that connects the Han and indigenous cultures by combining the emerging technologies of unmanned vehicles and IoT, so that Han Chinese students and indigenous students can recognize and understand each other’s culture during the course. It also helps indigenous students understand the discussion on the relationship between unmanned vehicles, their living environments, and their culture, to draw them closer to technology. Based on the computational thinking performance of students, the study constructed a circular computational thinking process, which facilitates thinking combined with the use of unmanned vehicles, so that rational technological performance can strengthen effective understanding and thinking. Consequently, students with affective dispositions can bolster their development in program understanding. During the implementation of the application plan, the learning performance of indigenous students in computational thinking, as well as their recognition and integrated understanding of multicultural education, was fully explored. This study focuses on a multi-ethnical learning environment and discusses students’ performance in computational thinking ability when using unmanned aerial vehicle technology, and the effects of integrating culturally responsive teaching on students’ cultural awareness and programming and logical thinking abilities. For the evaluation of computational thinking ability, this study refers to the definition of TISA, which includes the performance in the ability to understand a problem, problem-solving assessment, logical thinking assessment, and self-assessment of programming ability (Román-González et al., 2017). For the recognition and understanding of cultural response, we focus on whether the introduction of multicultural teaching materials has facilitated the understanding, recognition, and connection to cultures, and discuss whether the mediating effect of cultural response can induce Han Chinese and indigenous students to understand the problems of their respective cultures and identify possible solutions. The overall research model implemented is shown in Figure 1.

### 3.2. Computational thinking teaching with unmanned vehicles

Unmanned vehicles—that is, unmanned cars, UAV, unmanned boats, etc.—are a kind of machine system that can be automatically or remotely controlled. This study plans to use UAV for the introduction of course teaching. In the subsequent design and thinking project, the use of unmanned vehicles is selected according to the needs of students. UAV were used for military purposes when they were first introduced. With the recent development of IoT and AI, sensors, photographic devices, and even infrared lenses are added to the vehicles, so that they can be used in various industries. In addition to commercial applications, many courses have an introduction to learning about unmanned vehicles, including maker education courses that teach students to make UAV as per STEM education. Tello EDU supports the compilation of multiple programming languages, including python and scratch. It also supports external connections of IoT sensors and LED dot matrix displays to realize different application supports. UAV are controlled with programs according to the programming education in the corresponding course; the learning scenarios of computational thinking that are expected to be constructed include the circular challenge, door-type challenge, and color cards of different colors. After learning the basic programming concepts in the first half of the course, students will be able to practice program compilation in an exercising scenario, so that they can practice computational thinking through practical operations. With the aid of computational thinking approaches, we expect students to perform decomposition, pattern recognition, abstraction, and simplification, and use algorithms. The practice starts with attempts to transform basic problems, and students are asked to list the actions completed by the unmanned aerial vehicles, including take-off, landing, moving straight, left, or right, and taking photos. Thereafter, they will be asked to simplify and list the information and perform program compilation and practical operation exercises according to the actions listed. The practiced operations corresponding to the content of the course are shown in Table 1.

**Table 1:** Computational thinking learning course.

Lesson	Course content	Challenges for unmanned vehicle operations	Computational thinking
1	Variable design	Control the unmanned aerial vehicle to land after flying a specific distance	Break up the process actions of the unmanned aerial vehicle Discuss the mode of flying List the same actions Writing the program of unmanned aerial vehicles
2	Loop processing	Control the unmanned aerial vehicle to fly a specific number of circles or fly back and forth	Break up a single-loop action Discuss whether there are similar actions Attempt to simplify the actions Write the program for unmanned aerial vehicles
3	Judging the conditions	Look for the red circle with the unmanned aerial vehicle	Break up, explain, and complete the request of the challenge Discuss the identification of camera operation Simplify simple actions Write the program for unmanned aerial vehicles
4	Function	The unmanned aerial vehicle rotates in circles after locating the red circle	Break up the actions of seeking and circling Discuss the consistencies in seeking and circling Simplify the circling actions Write the program for unmanned aerial vehicles
5	Recursion	Search for the red and yellow devices in the designated order and repeat this three times	Break up, explain, and complete the request of the challenge Discuss the actions of seeking the colors Simplify the actions of the three cycles Write the program for unmanned aerial vehicles

### 3.3. Integration of culturally responsive teaching

Corresponding to the objectives of multicultural teaching, the main purpose of culturally responsive teaching is to establish a just educational environment for ethnic minorities by promoting the understanding of different cultures. Culturally responsive teaching implements practical multicultural teaching; it hopes to encourage students to become familiar with their own cultural history and teaching atmosphere by bridging multi-ethnic cultures, thereby improving the learning motivation and learning effectiveness of minority students. This study attempts to combine and integrate unmanned vehicles with culturally responsive teaching, introduce indigenous cultures and activities to the content of the course, and combine design thinking and empathy, so as to understand the needs of different ethnic groups and the impacts of introducing technology. The activities and designs are planned according to culturally responsive teaching. There are three main components in the plan: course design, teachers’ roles, and teaching strategies. Course design includes the integration of indigenous cultural content in an additional mode in response to the cultural backgrounds and experiences of indigenous students. Teachers’ roles include having relevant multicultural concepts, respect for diverse cultures, and awareness of equal education. Teaching strategies include offering corresponding evaluations and assessments for the learning modes of Han Chinese and indigenous students, using diverse evaluation methods. The additional cultural introduction is a common method for practicing cultural response teaching, which enables students to naturally acquire the corresponding cultural connotation by introducing cultural factors. Issues and applications related to Han Chinese and indigenous cultures were introduced to the teaching delivered by the course of this study; unmanned vehicles and IoT technology were used for the application and introduction of Han and indigenous cultures; and students were encouraged to reflect on and discuss how emerging technologies affect them. With the additional integration of indigenous cultures, while enhancing Han Chinese students’ understanding of indigenous cultures and encouraging them to explore indigenous issues in the process of course teaching, indigenous students could also reflect on how emerging technologies affect them by discussing their own issues, in order to improve their overall learning effectiveness and cultural awareness. The corresponding plans and arrangement of the additional integration of indigenous cultures are shown in Table 2.

**Table 2:** Corresponding plans and arrangement of the additional integration of indigenous cultures.

Teaching the operation of UAV	Introduction to additional integration of indigenous cultures	Student exercise	Design of the challenge
Introduction to the basic operation of unmanned aerial vehicles	The influence of hawks in tribal history and cultures, and relevant stories	Practice making cultural totems during the exercises involving the use of unmanned vehicles	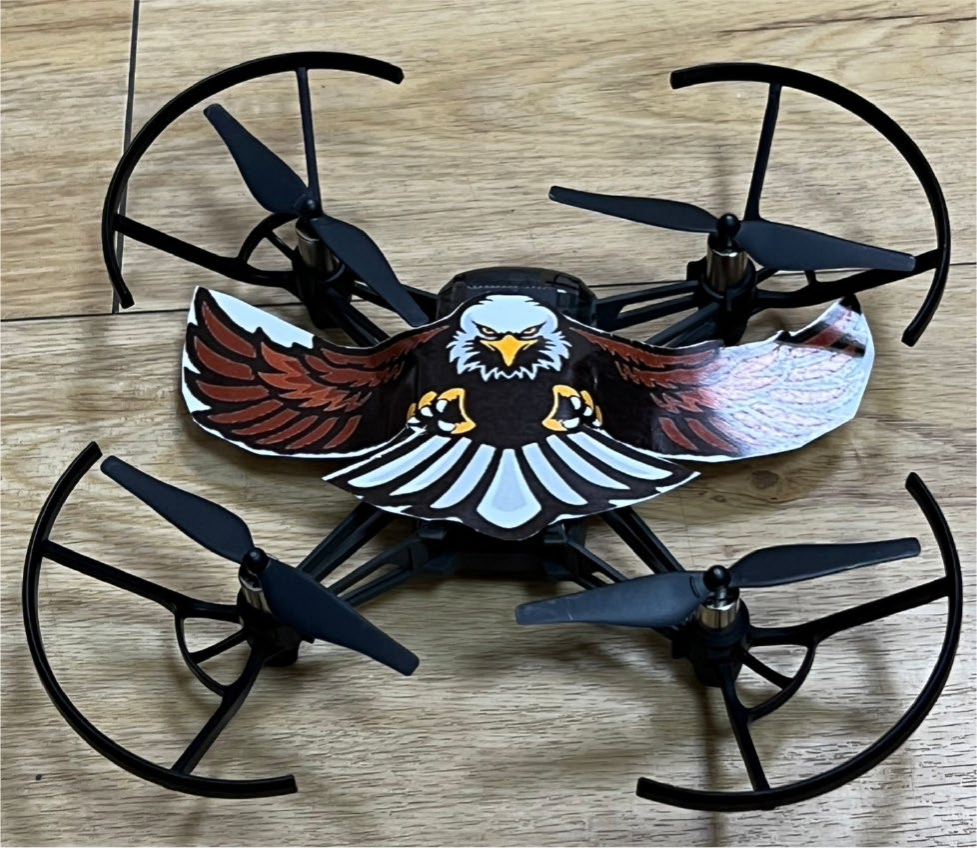
Controlling UAV during challenges	The course introduces the clothing and craft cultures of indigenous peoples: the clothing of indigenous men and women and its relations to festivals and cultures	Practice designing indigenous culture maps at the starting points and circles of challenges	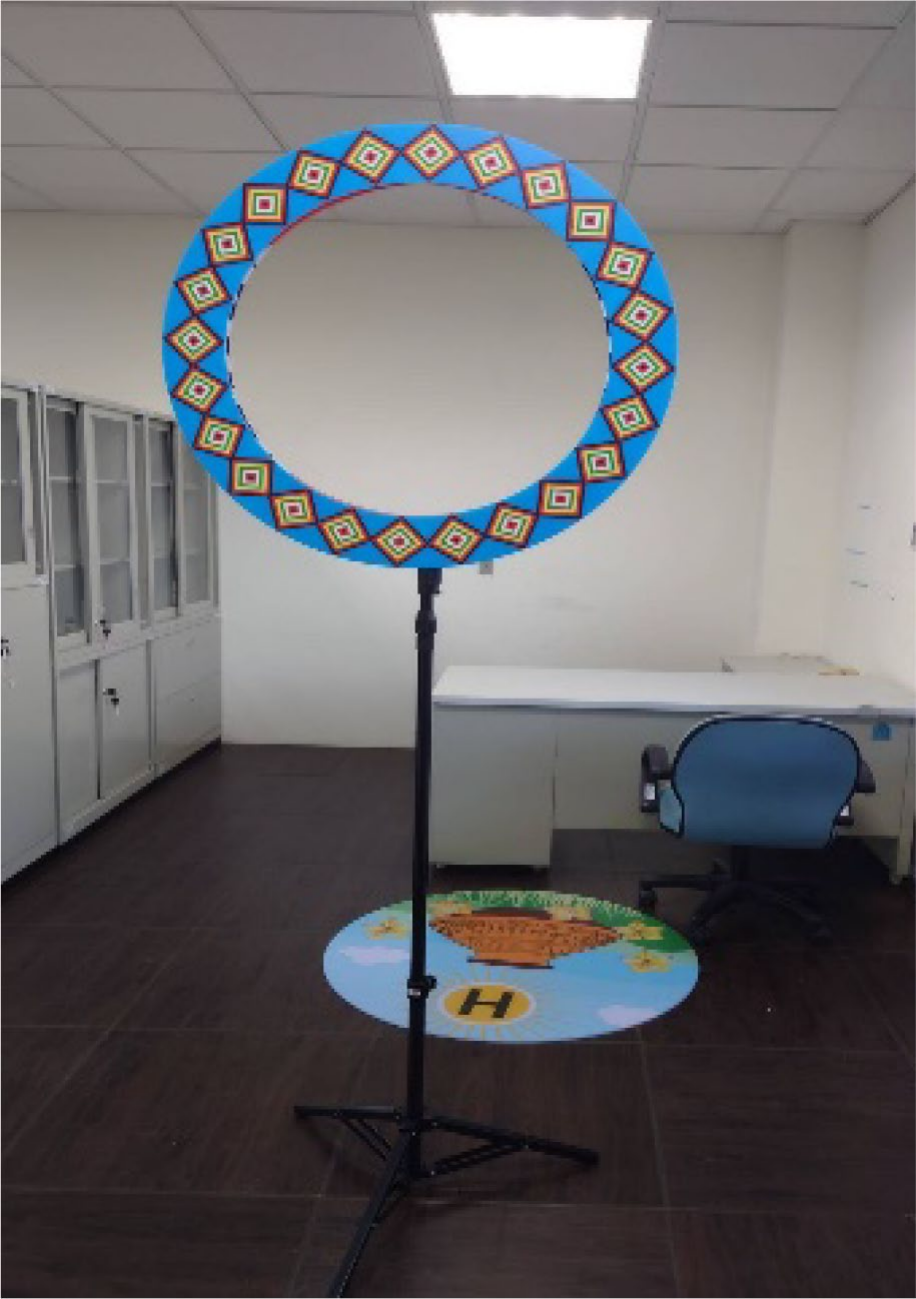
Control the flying	The course introduces the architectural history of indigenous tribes and tribal culture	Practice building tribal challenges and explaining the features of tribes	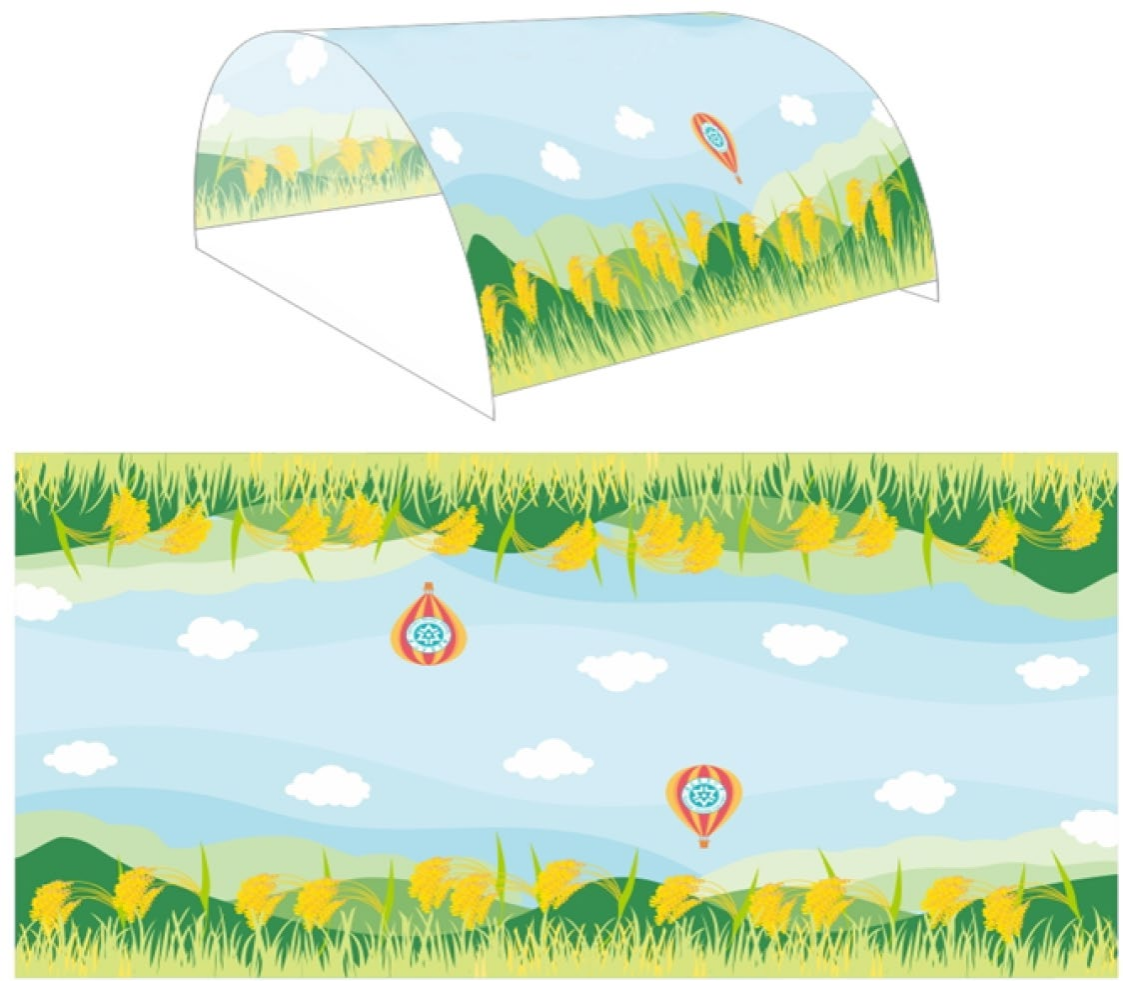
Automatic control of unmanned aerial vehicles	Introduction to the geographical location and cultural influence of tribes	Use of automatic control to locate indigenous scenes with the guidance of flags and cultural totems and lead the vehicles back to the tribal footholds	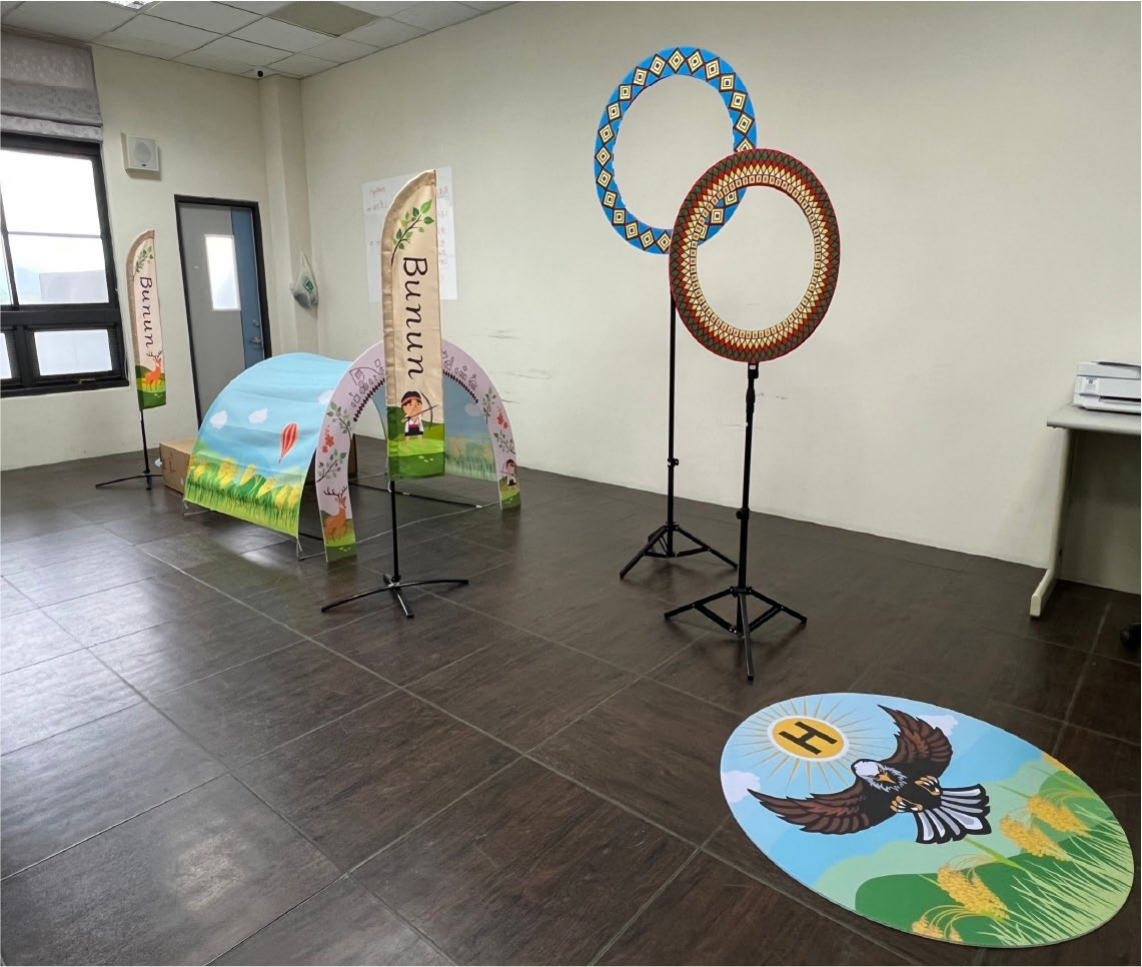

## 4. Experimental data collection and analysis results

The main purpose of this study was to establish a UAV-assisted teaching environment with the integration of culturally responsive teaching to test teaching strategies. The main approaches used include a “control-group pre-test and post-test design” of a “quasi-experimental design.” Junior and senior university students who majored in information engineering and had taken courses on IoT applications were invited to join the research project. Among them, 17 Han Chinese and six indigenous students were allocated to the traditional teaching group (control group), and 14 Han Chinese and eight indigenous students were allocated to the culturally responsive teaching group (experimental group)—45 students participated in the experimental research. The first 8 weeks of the experimental course were spent mainly on teaching basic programming computational thinking. After both groups had learned the basics of programming, the pro-test was conducted. Thereafter, a test of culturally responsive teaching was conducted on the experimental and control groups during the practice. In the last week, a post-test was conducted with tests on computational thinking and programming.

### 4.1. Measurement tools

In this study, we hope to analyze and discuss the students’ professional knowledge and cultural awareness and understanding through two major aspects: learning assessment and self-efficacy assessment. Three main measurement tools were used in this study:

- Bebras international challenge: The Bebras International Challenge on Informatics and Computational Thinking was employed in this study as test items to assess students’ computational thinking ability. The total score of the computational thinking test was 300 points (Dagienė and Futschek, 2008). This test is designed to determine whether students have problem thinking, programming skills, and logical performance in computational thinking.
- Program testing: This test is mainly a basic program test, which measures the effectiveness of students’ professional knowledge acquisition in program learning. The content of the test is program writing, loop description, and judgment logic program.
- Self-efficacy questionnaire: The self-efficacy questionnaire was designed to measure students’ cognitive effectiveness in cultural understanding and logical thinking skills. The factor loading, reliability, and validity of the questionnaire were verified, and two-way ANCOVA was used to analyze the information to confirm the relationship of different ethnic groups and teaching approaches with the results of different evaluation items of computational thinking and cultures.

### 4.2. Learning outcomes of multi-ethnic students with unmanned aerial vehicle-assisted culturally responsive teaching

The research explores students’ learning outcomes after computational thinking teaching under the influence of different teaching strategies. To eliminate the effects of the originally existing differences between the Han Chinese and indigenous students in their basic computational thinking ability, covariate analysis was used to analyze the effects on the computational thinking outcomes. Covariate analysis—a statistical control method—is often used in the analysis and application of data in the quasi-experimental research method, that is, using statistical means to eliminate errors that may affect the accuracy of the experiment. The results are shown in Table 3. The traditional teaching method implemented on the indigenous student group had a mean score of 153.743 and a standard deviation of 16.432, adjusted for covariates. The culturally responsive teaching method applied on the indigenous student group had a mean score of 200.514 and a standard deviation of 18.323, adjusted for covariates. The traditional teaching method used on the Han Chinese students had a mean score of 160.491 and a standard deviation of 31.588, adjusted for covariates. The culturally responsive teaching method applied on the Han Chinese students had a mean score of 200.514 and a standard deviation of 18.323, adjusted for covariates.

**Table 3:** Summary of the descriptive statistical analysis by covariate analysis.

Ethnic	Teaching method	*N*	*M*	*SD*	Mean after adjustment
Indigenous	Traditional	6	145.000	16.432	153.743
	Cultural response	8	187.500	18.323	200.514
Han	Traditional	17	161.176	24.719	160.491
	Cultural response	14	211.429	31.588	201.078

The results of Levene’s test for equality of error variances are shown revealed in Levene’s homogeneity of variance test, *F*(3, 41) = 2.326, *p* = 0.089 > 0.05. The hypothesis of error variances should be accepted—that is, the dependent variables of the Han Chinese and indigenous student groups have homogeneity of variance. In Table 4, the test of homogeneity of the within-group regression coefficient shows that the *F* value between groups and teaching methods is 0.946, whereas *p* = 0.428, which reveals that the within-group regression coefficients are homogenous and that a two-factor covariate analysis is a suitable approach.

**Table 4:** Test of homogeneity of the within-group regression coefficient.

Source of variance	SS	*df*	MS	*F*	*p*
Between groups (regression coefficients)	738.707	3	246.236	0.946	0.428
Within group (error)	9,628.368	37	260.226		
Whole sample	10,367.075	40			

**Table 5:** Covariate test analysis.

Source of variance	SS	*df*	MS	*F*	*p*	*Post hoc* comparison
Ethnic (A)	109.403	1	109.403	0.422	0.520	
Teaching method (B)	18,010.238	1	18,010.238	69.490	0.000	The traditional method (177.505) is significantly lower than the culturally responsive method (181.162)
A × B	88.103	1	88.103	0.340	0.563	
Error	10,367.075	40	259.177			

In Table 5, the results of the overall covariate analysis show that the *P* value of the AxB interaction effect is 0.563, so there is no two-factor interaction effect, and the *post hoc* comparative analysis can be directly conducted. According to the post-hoc analysis results, in the Bebras test results, the ethnic group factor is insignificant while comparing the teaching methods; that of the traditional method (177.505) is significantly lower than that of the culturally responsive method (181.162).

### 4.3. Programming ability of multi-ethnic students with unmanned aerial vehicle-assisted culturally responsive teaching

A comparison of the midterm and final program tests were conducted to see if culturally responsive teaching methods had an impact on students’ program learning performance in a diverse group. According to the results in Table 6, the covariance-adjusted mean of traditional instruction for the indigenous student group was 45.265 with a standard deviation of 24.221; the covariance-adjusted mean of culturally responsive instruction for the indigenous student group was 46.380 with a standard deviation of 15.964; the covariance-adjusted mean of traditional instruction for Han students was 43.786 with a standard deviation of 26.761; and the covariance-adjusted mean of culturally responsive instruction for Han students was 41.786 with a standard deviation of 26.761. The covariance-adjusted mean of culturally responsive teaching method was 41.929, with a standard deviation of 15.711.

**Table 6:** Summary of the descriptive statistical analysis by covariate analysis.

Ethnic	Teaching method	*N*	*M*	*SD*	Mean after adjustment
Indigenous	Traditional	6	38.333	24.221	45.265
	Cultural response	8	50.375	15.964	46.380
Han	Traditional	17	48.176	26.761	43.786
	Cultural response	14	37.286	15.711	41.929

The results of Levene’s test for equality of error variances are shown that reveals in Levene’s homogeneity of variance test, *F*(3, 41) = 0.923, *p* = 0.438 > 0.05. The hypothesis of error variances should be accepted. The homogeneity of variance between the Han and the indigenous student groups on the program test. The test of homogeneity of the within-group regression coefficient shows that the F value between groups and teaching methods is 1.100, whereas *p* = 0.362, which reveals that the within-group regression coefficients are homogenous and that a two-factor covariate analysis is a suitable approach (Table 7).

**Table 7:** Test of homogeneity of the within-group regression coefficient.

Source of variance	SS	*df*	MS	*F*	*p*
Between groups (regression coefficients)	441.841	3	147.280	1.100	0.362
Within group (error)	4955.997	37	133.946		
Whole sample	5397.838	40			

The results of the overall covariate analysis are shown in Table 8. The *P* value of the interaction in AXB was 0.705, so there was no two-factor interaction, which could be directly analyzed in the *post hoc* comparative analysis, and the results of the *post hoc* analysis showed that the results were not significant in the group factor for program ability and not significant in the pedagogy.

**Table 8:** Covariate test analysis.

Source of variance	SS	*df*	MS	*F*	*p*
Ethnic (A)	83.226	1	83.226	0.617	0.437
Teaching method (B)	1.304	1	1.304	0.010	0.922
A × B	19.622	1	19.622	0.145	0.705
Error	5397.838	40	134.946		

### 4.4. Cultural respect of multi-ethnic students with unmanned aerial vehicle-assisted culturally responsive teaching

Culturally responsive teaching has different scales for considering different elements such as environment, teachers, and materials. Gay and Howard (2000) focus on the power of concern, culture, and communication in the classroom, racial and cultural diversity in curriculum content, and cultural congruity in teaching and learning. Wlodknowski and Ginsberg (1995) reviewed the environment, instruction, and instructor quality together, including respecting cultural differences, enhancing motivation, creating equitable learning environments, teaching across cultures, and promoting equity and justice. In this study, we focused on the impact of teaching strategies and materials design on students’ perceptions, so we used the cultural respect construct proposed by Gay and Howard (2000) to create a relevant questionnaire. The results of the analysis based on the questionnaire are shown in Table 9. The covariance-adjusted mean of traditional teaching method for the indigenous group was 23.177, with a standard deviation of 0.516; the covariance-adjusted mean of culturally responsive teaching method for the indigenous group was 23.894, with a standard deviation of 0.463; the covariance-adjusted mean of traditional teaching method for Han Chinese students was 20.942, with a standard deviation of 1.967; the covariance-adjusted mean of culturally responsive teaching method for Han Chinese students was 24.340, with a standard deviation of 1.967. The covariance-adjusted mean was 24.340 and the standard deviation was 1.899.

**Table 9:** Summary of the descriptive statistical analysis.

Ethnic	Teaching method	*N*	*M*	*SD*	Mean after adjustment
Indigenous	Traditional	6	24.333	0.516	23.177
	Cultural response	8	24.750	0.463	23.894
Han	Traditional	17	20.647	1.967	20.942
	Cultural response	14	23.714	1.899	24.340

The results of Levene’s test for equality of error variances are shown that reveals in Levene’s homogeneity of variance test, *F*(3, 41) = 0.923, *p* = 0.438 > 0.05. The hypothesis of error variances should be accepted. The homogeneity of variance between the Han and the indigenous student groups on the program test. The test of homogeneity of the within-group regression coefficient shows that the *F* value between groups and teaching methods is 0.144, whereas *p* = 0.933, which reveals that the within-group regression coefficients are homogenous and that a two-factor covariate analysis is a suitable approach (Table 10).

**Table 10:** Test of homogeneity of the within-group regression coefficient.

Source of variance	SS	*df*	MS	*F*	*p*
Between groups (regression coefficients)	1.136	3	0.379	0.144	0.933
Within group (error)	97.121	37	2.625		
Whole sample	98.257	40			

The results of the overall covariate analysis are shown in Table 11. The *P* value of the interaction in AXB was 0.012 < 0.05, so there was a two-factor interaction situation, which could not be directly analyzed in a *post hoc* comparative analysis, and further covariate analysis of the main effect alone was required.

**Table 11:** Covariate test analysis.

Source of variance	SS	*df*	MS	*F*	*p*
Ethnic (A)	2.998	1	2.998	1.221	0.276
Teaching method (B)	37.476	1	37.476	15.256	0.001
A × B	17.034	1	17.034	6.934	0.012
Error	98.257	40	2.456		

In the statistical analysis of the main effects alone, as shown in Table 12, it was found that the performance of cultural respect for the pedagogy was insignificant in the ethnic group factor. Among the pedagogical factors, it was not significant for the indigenous ethnic groups but significant for the Han students. Based on the *post hoc* performance, it was found that the performance of cultural respect was significantly lower than that of culturally responsive pedagogy for the Han students using traditional teaching methods.

**Table 12:** Statistical analysis of the main effects.

Source of variance	SS	*df*	MS	*F*	*p*	*Post-hoc* comparison
Ethnic (A)						
Traditional (b1)	10.004	1	10.004	3.672	0.070	
Cultural Response (b2)	0.226	1	0.226	0.098	0.758	
Teaching method (B)						
Indigenous (a1)	0.539	1	0.539	2.113	0.174	
Han (a2)	83.830	1	83.830	24.713	0.000	The traditional method (20.491) is significantly lower than the culturally responsive method (23.904)
Error	98.257	40	2.456			

### 4.5. Logical thinking of multi-ethnic students with unmanned aerial vehicle-assisted culturally responsive teaching

Logical thinking is a key indicator of learning in education today, as it allows learners to understand problems effectively and to identify patterns (Al-Thallab, 2021). In this study, a questionnaire was administered to determine the students’ perceptions of logical thinking after learning through the computational thinking approach. According to the results in Table 13, the covariance-adjusted mean of the traditional teaching method for the Native group was 20.266 with a standard deviation of 3.189; the covariance-adjusted mean of culture responsive teaching method for the Native group was 23.426 with a standard deviation of 2.264; the covariance-adjusted mean of traditional teaching method for Han students was 20.810 with a standard deviation of 1.770; and the covariance-adjusted mean of culture-responsive teaching method for Han students was 1.770. The covariance-adjusted mean of response method was 23.516, with a standard deviation of 1.899.

**Table 13:** Summary of the descriptive statistical analysis.

Ethnic	Teaching method	*N*	*M*	*SD*	Mean after adjustment
Indigenous	Traditional	6	20.167	3.189	20.266
	Cultural response	8	23.625	2.264	23.426
Han	Traditional	17	20.588	1.770	20.810
	Cultural response	14	23.714	1.899	23.516

The results of Levene’s test for equality of error variances are shown, which reveals that in Levene’s homogeneity of variance test, *F*(3, 41) = 0.537, *p* = 0.660 > 0.05. The hypothesis of error variances should be accepted. The homogeneity of variance between the Han and the indigenous student groups on the program test. In Table 14, the test of homogeneity of the within-group regression coefficient shows that the *F* value between groups and teaching methods is 1.016, whereas *p* = 0.397 > 0.05, which reveals that the within-group regression coefficients are homogenous and that a two-factor covariate analysis is a suitable approach.

**Table 14:** Test of homogeneity of the within-group regression coefficient.

Source of variance	SS	*df*	MS	*F*	*p*
Between groups (regression coefficients)	11.769	3	3.923	1.016	0.397
Within group (error)	142.876	37	3.862		
Whole sample	154.646	40			

The results of the overall covariate analysis is shown in Table 15. The *P* value of the A×B interaction effect is 0.724 > 0.05, so there is no two-factor interaction effect, and the post-hoc comparative analysis can be directly conducted. According to the post-hoc analysis results, in the results, the ethnic group factor is insignificant while comparing the teaching methods; that of the traditional method (21.886) is significantly lower than that of the culturally responsive method (22.203).

**Table 15:** Covariate test analysis.

Source of variance	SS	*df*	MS	*F*	*p*	*Post-hoc* comparison
Ethnic (A)	0.950	1	0.950	0.246	0.623	
Teaching method (B)	78.238	1	78.238	20.237	0.000	The traditional method (21.886) is significantly lower than the culturally responsive method (22.203)
A × B	0.490	1	0.490	0.127	0.724	
Error	154.646	40	3.866			

## 5. Discussion and conclusion

This study uses unmanned vehicles and IoT technology to introduce culturally responsive teaching, so that Han Chinese and indigenous students in Taiwan can use this technology to consider their respective cultural problems based on their different cultures and living environments and attempt to devise suitable solutions with programming. It also attempts to establish a circular operation thinking mode to explore the performance of Han Chinese and indigenous students in their respective programming abilities, as well as grasp the effects of flipped learning strategies on the motivation and effectiveness of learning programming. The study’s observations are discussed below.

### 5.1. Learning outcomes of multi-ethnic students after culturally responsive teaching and unmanned aerial vehicle-assisted unmanned vehicles teaching

The main purpose of this study was to identify a suitable method for teaching computational thinking to multi-ethnic groups and discuss the factors related to the overall learning effectiveness through culturally responsive teaching. The Bebras International Challenge on Informatics and Computational Thinking, which generates students’ assessment results, was used in this study to test students’ computational thinking ability. The study reveals that the ethnic group factor was not an influencing factor, whereas the culturally responsive teaching method introduced with unmanned vehicle teaching impacted the results. Further evaluation demonstrated that the introduction of cultural response teaching has a higher impact on the learning effectiveness of indigenous students. This is probably because the cultural teaching and guidance in the course helped improve indigenous students’ willingness to study, and cultural identity and cultural equality increased their engagement in learning relevant knowledge and skills.

### 5.2. The effect of culturally responsive teaching and unmanned aerial vehicle-assisted computational thinking teaching on the performance of multi-ethnic students’ programming ability

Block-based and python programming languages were used in the course delivered by this study to control the flying of unmanned vehicles. The overall purpose was to help learners find relevant calculation methods and information programming designs corresponding to practical problems; many of the teaching objectives also regarded the results of programming design as the final evaluation criteria. However, as indigenous students are usually influenced by prior knowledge and the environment, their performance in writing programs and learning algorithms usually lags behind that of other students. We originally expected to enhance the Han Chinese and indigenous students’ learning of unmanned vehicle programs in a culturally responsive environment, but the experiment results showed that the learning performance of programs was not significant in all ethnic groups and for all teaching methods. The interviews showed that, in the process of learning, as the unmanned vehicles could be directly controlled or operated with a repeated test approach, the students were more inclined to directly operate and verify the operation of the unmanned vehicles, so that all results of the performance of programming ability were insignificant.

### 5.3. The effect of culturally responsive teaching and unmanned aerial vehicle-assisted computational thinking teaching on the performance of multi-ethnic students’ cultural respect

In a learning environment that is multi-ethnic friendly, cultural respect is a key factor in establishing holistic culturally responsive teaching. By introducing challenges and themes related to indigenous cultures for unmanned vehicle operation, this study encouraged Han Chinese and indigenous students to collaborate in comprehending and designing the implementation of unmanned vehicle operation, and jointly verify the results. The questionnaire survey shows that, in terms of cultural respect performance, ethnic groups and culturally responsive teaching methods are mutually influential. The simple main effect test showed that, as the Han Chinese students who received traditional teaching had never been exposed to relevant multicultural learning, their performance on multicultural cognition and respect cognition was significantly lower than that of their counterparts who had received culturally responsive teaching. However, as the indigenous students had been trained on cultural cognition and understanding since their childhood, a certain level of cultural respect was displayed in both groups, regardless of whether they received traditional or culturally responsive teaching.

### 5.4. The effect of culturally responsive teaching and unmanned aerial vehicle-assisted computational thinking teaching on the performance of multi-ethnic students’ logical thinking

Studies have shown that unmanned vehicles are effective in improving students’ logical thinking abilities. This study further explored their effects on logical thinking for different ethnic groups and teaching methods. This study revealed that the performance of logical thinking in the traditional learning method was significantly lower than that in the culturally responsive teaching method. According to the post-hoc interviews, the key reason was that in the culturally responsive teaching process, the challenges were designed to integrate indigenous cultures, instead of simple flying challenges. Additionally, in the process of designing the flying challenges and the display of thematic scenarios by themselves, the students could better understand spatial logic and be better trained in logical thinking.

According to the results of this study, using unmanned vehicles to introduce culturally responsive teaching for exploration and learning can strengthen the joint thinking of Han Chinese and indigenous students on multicultural issues, strengthen Han Chinese students’ understanding and empathy of indigenous cultures, and reduce the distance between emerging technology applications and indigenous cultures, so that the former can facilitate the influence and learning identity related to cultures. The experiment also verified that under the guidance of cultures, in addition to helping indigenous students enhance their learning effectiveness relevant to computational thinking, the adjustment of course learning designs can also improve students’ motivation and effectiveness in learning programming. Moreover, in the process of learning, through mutual aid and cooperation between the Han Chinese and indigenous peoples, their understanding of each other’s culture can be enhanced. Based on such a teaching design plan, we can also discuss the Han Chinese and indigenous students’ performance in computational thinking ability.

## Data availability statement

The raw data supporting the conclusions of this article will be made available by the authors, without undue reservation.

## Author contributions

The author confirms being the sole contributor of this work and has approved it for publication.

## Funding

This research was supported by a grant from National Science and Technology Council Project NSTC 110-2511-H-143-002-.

## Conflict of interest

The author declares that the research was conducted in the absence of any commercial or financial relationships that could be construed as a potential conflict of interest.

## Publisher’s note

All claims expressed in this article are solely those of the authors and do not necessarily represent those of their affiliated organizations, or those of the publisher, the editors and the reviewers. Any product that may be evaluated in this article, or claim that may be made by its manufacturer, is not guaranteed or endorsed by the publisher.
